# Galectin-3 Impairment of MYCN-Dependent Apoptosis-Sensitive Phenotype Is Antagonized by Nutlin-3 in Neuroblastoma Cells

**DOI:** 10.1371/journal.pone.0049139

**Published:** 2012-11-09

**Authors:** Veronica Veschi, Marialaura Petroni, Beatrice Cardinali, Carlo Dominici, Isabella Screpanti, Luigi Frati, Armando Bartolazzi, Alberto Gulino, Giuseppe Giannini

**Affiliations:** 1 Department of Experimental Medicine, Sapienza University, Rome, Italy; 2 Department of Molecular Medicine, Sapienza University, Rome, Italy; 3 Institute of Cell Biology and Neurobiology, National Research Council, Monterotondo Scalo, Italy; 4 Department of Pediatrics, Sapienza University, Rome, Italy; 5 School of Reproductive and Developmental Medicine, Liverpool University, Liverpool, United Kingdom; 6 Experimental Pathology Laboratory, S. Andrea Hospital, Rome, Italy; 7 Cancer Center Karolinska (CCK) R8∶04, Karolinska Hospital, Stockholm, Sweden; Complutense University, Spain

## Abstract

MYCN amplification occurs in about 20–25% of human neuroblastomas and characterizes the majority of the high-risk cases, which display less than 50% prolonged survival rate despite intense multimodal treatment. Somehow paradoxically, MYCN also sensitizes neuroblastoma cells to apoptosis, understanding the molecular mechanisms of which might be relevant for the therapy of MYCN amplified neuroblastoma. We recently reported that the apoptosis-sensitive phenotype induced by MYCN is linked to stabilization of p53 and its proapoptotic kinase HIPK2. In MYCN primed neuroblastoma cells, further activation of both HIPK2 and p53 by Nutlin-3 leads to massive apoptosis in vitro and to tumor shrinkage and impairment of metastasis in xenograft models. Here we report that Galectin-3 impairs MYCN-primed and HIPK2-p53-dependent apoptosis in neuroblastoma cells. Galectin-3 is broadly expressed in human neuroblastoma cell lines and tumors and is repressed by MYCN to induce the apoptosis-sensitive phenotype. Despite its reduced levels, Galectin-3 can still exert residual antiapoptotic effects in MYCN amplified neuroblastoma cells, possibly due to its specific subcellular localization. Importantly, Nutlin-3 represses Galectin-3 expression, and this is required for its potent cell killing effect on MYCN amplified cell lines. Our data further characterize the apoptosis-sensitive phenotype induced by MYCN, expand our understanding of the activity of MDM2-p53 antagonists and highlight Galectin-3 as a potential biomarker for the tailored p53 reactivation therapy in patients with high-risk neuroblastomas.

## Introduction

Neuroblastoma (NB), the most common extracranial solid tumor of childhood, originates from the neural crest precursors involved in the development of the adrenal medulla and paraspinal sympathetic ganglia. Although children affected with NB might undergo spontaneous or therapy-induced regression, less than 50% of the high-risk patients experience long-term survival, despite intense multimodal treatment. Together with clinical and pathological features (i.e., age at diagnosis, stage, tumor grade, histology and DNA ploidy) MYCN amplification (MNA) contributes to the identification of high-risk patients [1] and represents one of the best independent markers of adverse outcome and very poor survival [2], [3]. MYCN belongs to the MYC family of transcription factors and can affect the expression of a number of genes driving cell cycle progression, cell metabolism, invasion and angiogenesis [4]. Targeting its expression to the neural crests of transgenic mice results in NB tumor development [5], highlighting the impact of this protein on neuroblastic cell carcinogenesis. Furthermore, MNA NB cells are addicted to MYCN and its depletion profoundly affects their survival, proliferation and differentiation *in vitro* and *in vivo* [6], [7].

**Figure 1:**
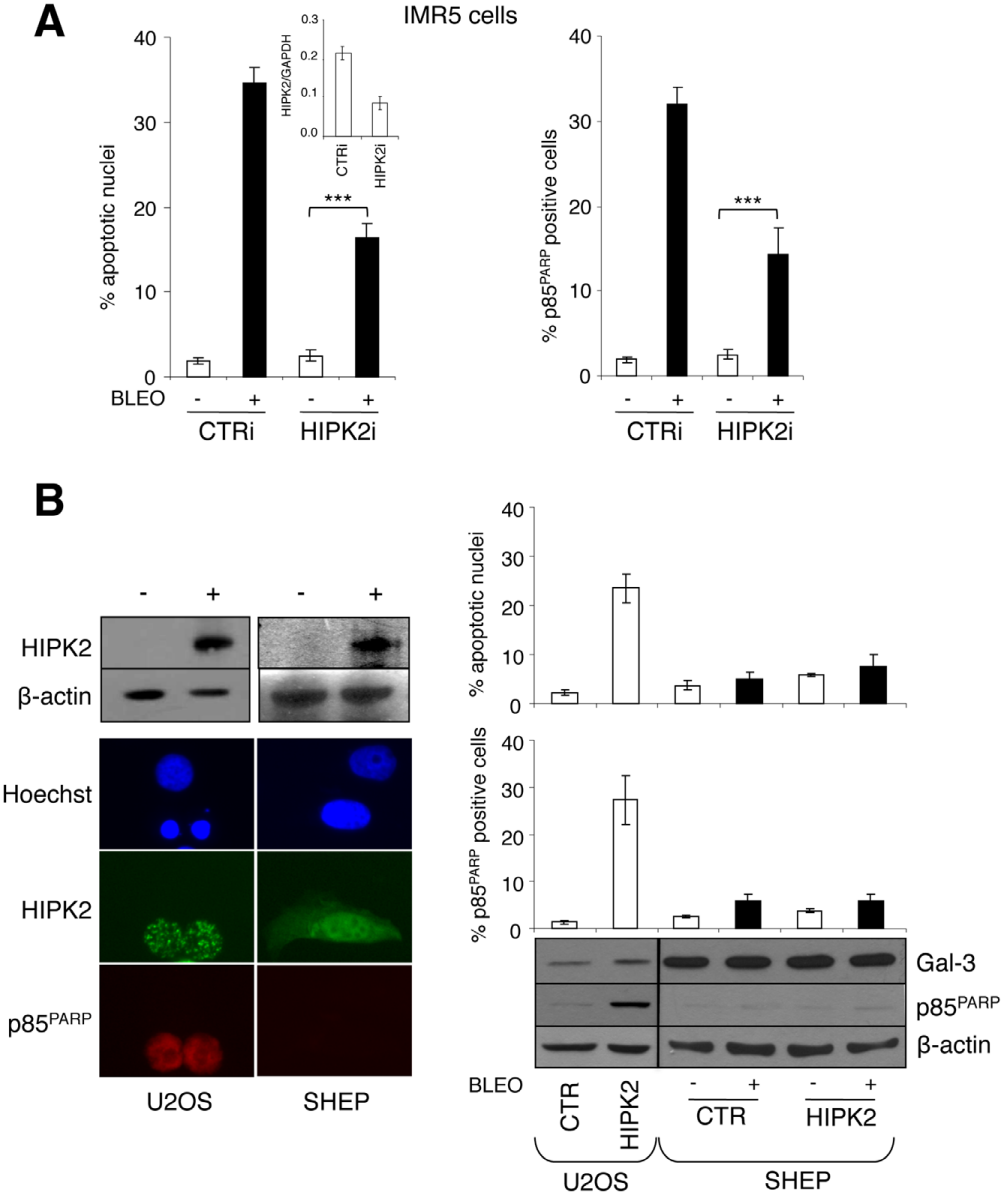
Exogenous HIPK2 expression is not sufficient to induce apoptosis or sensitizes MNSC cells to DNA damaging drugs. A , HIPK2 knock-down was achieved by transient transfection with sh-RNAi in MNA IMR-5 cells and was measured by Q-RT-PCR (inset). Analysis of bleomycin (5µg/ml) induced apoptosis is shown as percentage of apoptotic nuclei and cells positive for p85 cleaved fragment of the PARP protein (p85^PARP^). Significant differences in apoptosis fold induction were obtained between HIPK2i and CTRi transfected cells (***p<0.0001) **B**, Cell transfection with HIPK2 (+) but not empty vector (−) caused apoptosis in the U2OS osteosarcoma cells as indicated by the appearance of apoptotic nuclei and/or positive staining for p85^PARP^ (left panel), but failed to induce apoptosis and to sensitize MNSC SHEP neuroblastoma cells to bleomycin treatment (percentage of the apoptotic cells are given in the graphs in the right panel). The immunoblot (lower right panel) shows the accumulation of the indicated proteins in HIPK2 transfected and/or bleomycin treated cell extracts.

Somehow paradoxically, MYCN overexpression might also lead to apoptosis and/or sensitize cells to death [8], [9], [10]. Indeed, MNA NBs are sensitive to chemo- or radiotherapy at diagnosis, and MNA or MYCN overexpressing NB cell lines are more sensitive to apoptosis induced by DNA damaging agents as compared to MYCN single copy (MNSC) cells [11], [12]. Studying this paradox is likely to uncover molecular mechanisms potentially relevant for the therapy of MNA NBs. Indeed, by these means several groups, including ours, highlighted the p53 pathway as a potential target for MNA NB therapy [13], [14], [15].

p53 is a master oncosuppressor protein protecting cells from genetic instability and tumor development by inducing cell cycle arrest or apoptosis in response to cellular stress and DNA damage [16]. Not surprisingly, p53 is mutated in over 50% of human cancers. In sharp contrast, human NBs are almost invariably p53 wild type at diagnosis [17], with the proapoptotic pathways downstream of p53 intact and recruitable by cytotoxic drugs to induce p53-dependent cell death in NB experimental models [18], [19]. p53 is actively involved in the apoptosis-sensitive phenotype induced by MYCN. Indeed, MYCN increases p53 transcription [20] and it also induces stabilization of the p53 protein and its proapoptotic kinase HIPK2 via an oncogene-dependent DNA damage response (DDR) [13]. The high levels of mitosis/karyorrhexis and the initial responses to the induction chemotherapy shown by the majority of newly diagnosed MNA NBs are consistent with the integrity of the HIPK2-p53 axis observed in primary NBs [13]. Despite this, however, MNA NBs undergo a fast and lethal progression in most instances, and this might be related to genetic or functional inactivation of p53 [21]. Supporting this hypothesis, p53 mutation rate rises up to 15% in relapsed or previously treated NBs [22] and treatment of NB mouse xenografts with the p53 reactivating drug Nutlin-3 (Nut-3) leads to tumor shrinkage and impairment of metastasis [15]. Also consistent with a particular role of the HIPK2-p53 pathway in MYCN-dependent apoptosis, restoring p53 function by p53-MDM2 antagonists seems particularly effective in MNA and MYCN overexpressing NB cells [13], [14] where a further induction of the HIPK2 kinase specifically occurs upon treatment with these drugs [13]. Since this approach promises to be an interesting opportunity for the treatment of high-risk MNA NBs, a better understanding of the molecular mechanisms governing the MYCN-dependent apoptosis-sensitive phenotype and the regulation of cell death following p53 reactivation urges in the perspective of a tailored p53 reactivation therapy of NB.

Searching for candidates that could be involved in the apoptosis-sensitive phenotype induced by MYCN, we focused our attention on Galectin-3 (Gal-3). Gal-3 is a ß-galactoside-binding lectin involved in a variety of biological processes including pre-mRNA processing, cell cycle progression, cell adhesion, angiogenesis and apoptosis [23]. These different biological activities appear to be cell type specific and strictly linked to its heterogeneous subcellular localization pattern [23], [24], [25], [26], [27]. Gal-3 is frequently overexpressed in cancer cells where it has been linked to resistance to drug-induced cell death. Indeed, Gal-3 may interact with and stabilize the mitochondrial membrane, likely via its Bcl-2 homology domain-1 (NWGR domain), leading to the inhibition of cyctochrome c release and impairment of apoptosis [28], [29]. Furthermore, several evidences indicate that Gal-3 is connected to the HIPK2-p53 circuitry [30], [31], [32].

Here we report that MYCN represses Gal-3 as a part of its apoptosis-sensitive phenotype. In MNA cells, however, along with Gal-3 reduced levels of expression, we uncovered a distinct subcellular localization pattern compatible with the more aggressive behavior of these tumors. Finally, we show that MDM2-p53 antagonists, such as Nut-3, repress Gal-3 and this is required for their full activity on MNA NB tumors.

**Figure 2:**
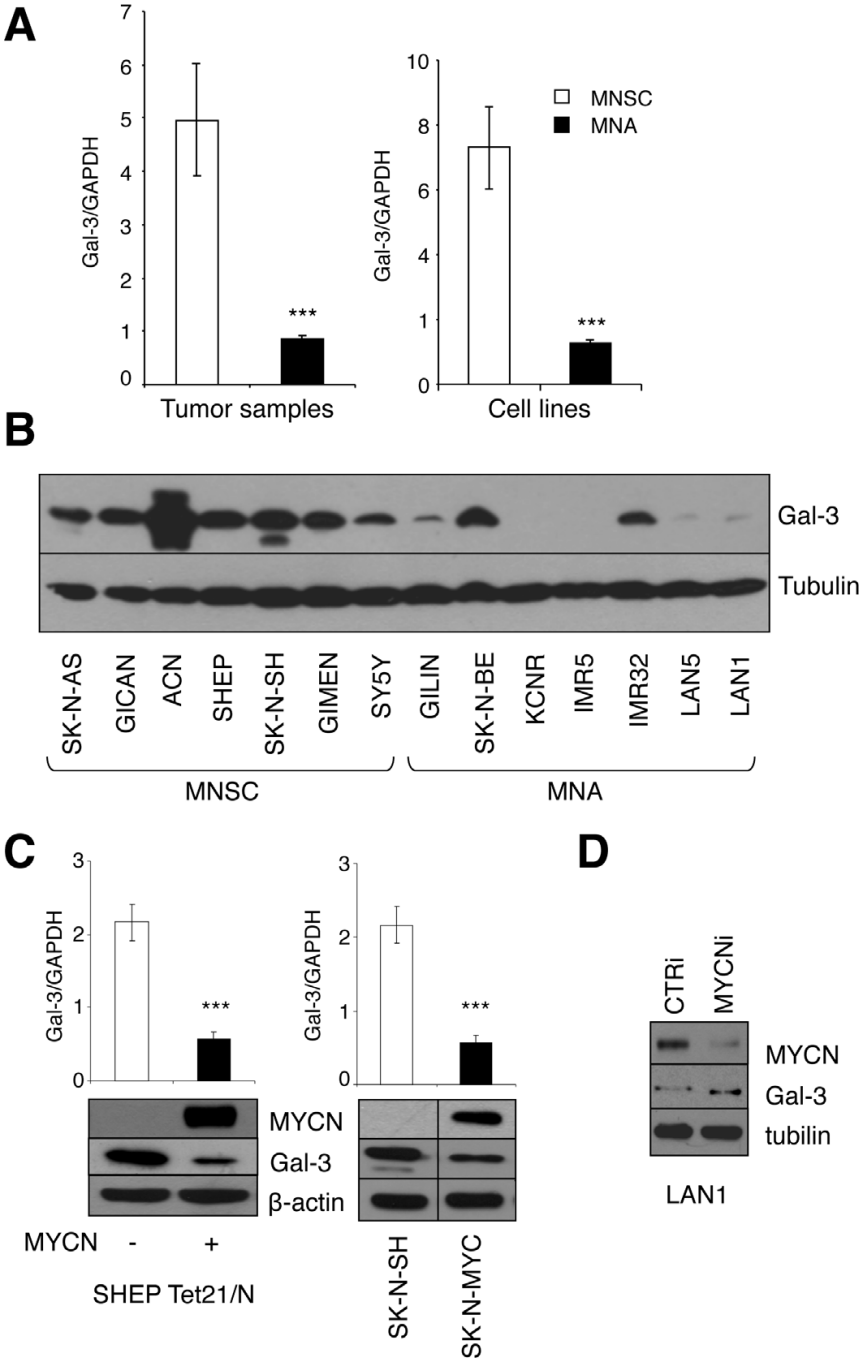
MYCN regulates Galectin-3 expression. A , Average Gal-3 mRNA expression (+/− standard deviation) in MNSC and MNA NB tumor samples and NB cell lines measured by Q-RT-PCR (***p<0.0001). Raw data are reported in Fig. S1. **B**, Immunoblot of Gal-3 protein expression in MNSC and MNA NB cells. **C**, Immunoblot (lower panels) and Q-RT-PCR analysis (upper panels) of Gal-3 expression in SHEP Tet21/N MYCN inducible cells and SK-N-MYC compared to parental SK-N-SH cells (***p<0.0001). **D**, MYCN knock-down was achieved via transient transfection with RNAi duplexes in MNA LAN1 cells and the expression of the indicated proteins was investigated by immunoblot.

## Materials and Methods

### Cell Lines and Culture Conditions

Human NB cells were obtained as follows. GICAN, ACN, GIMEN, GILIN and IMR5 were acquired from Banca Biologica and Cell Factory (Genoa, Italy; www.iclc.it); SK-N-BE and IMR32 from European Collection of Cell Cultures, (Porton Down, UK; www.ecacc.org.uk); LAN5 from Deutsche Sammlung Von Mikroorganismen und Zellkulturen (Braunschweig, Germany; www.dsmz.de). LAN1 cells [33] were a kind gift of Dr. Nicole Gross, Department of Pediatrics, University Hospital, Lausanne, Switzerland; SK-N-AS, SK-N-SH, SY5Y, KCNR [34] were a kind gift of Dr. Carol J. Thiele, CMBS, NCI, Bethesda, MD; SH-EP Tet21/N cells [35], received from Dr. Schwabb, DKFZ, Heidelberg, Germany were cultured and validated for MYCN inducibility as reported [35]. All cells were grown in standard conditions and validated by genetic search of MYCN amplification or other genetic aberrations. Cultured cells were currently tested for mycoplasma infection as reported [36].

### Apoptosis Assays and Gal-3 Detection in Immunofluorescence

Subconfluent cells were treated with either bleomycin, Casein Kinase 1 (CK1) inhibitor D4476 (Calbiochem, Darmstadt, Germany), adriamycin, cis-platin, Nut-3 (Sigma-Aldrich, St. Louis, MO, USA) for 24 hours (except IMR5 cells, 10 hours). Cell death was measured by tripan blue exclusion test. For the analysis of nuclear morphology, cells were fixed in 4% formaldehyde/PBS for 10 minutes, permeabilized in 0.25% Triton/PBS for 10 minutes, counterstained with 1 mg/ml Hoechst 33258, and mounted in PBS/50% glycerol. For the immunofluorescent analysis of the p85 cleaved fragment of the PARP protein (p85^PARP^), cells were fixed and permeabilized as above, blocked in 5% BSA, 3% goat serum in PBS for 1 hour and incubated ON with the primary antibody (Ab), followed by secondary Ab incubation, counterstained and mounted as above. For all three apoptosis assays, at least 200 cells/sample were counted in duplicate experiments. Each experiment was performed at least three times. Statistical analysis was performed by a standard two-tailed Student’s *t* test.

For the immunofluorescent analysis of Gal-3, cells were fixed and permeabilized as above and incubated ON with primary Ab, followed by secondary FITC-conjugated Ab incubation. For the MitoTracker assay (Invitrogen, Molecular Probes, San Diego, CA), cells were treated according to the manufacturer’s instructions.

**Figure 3:**
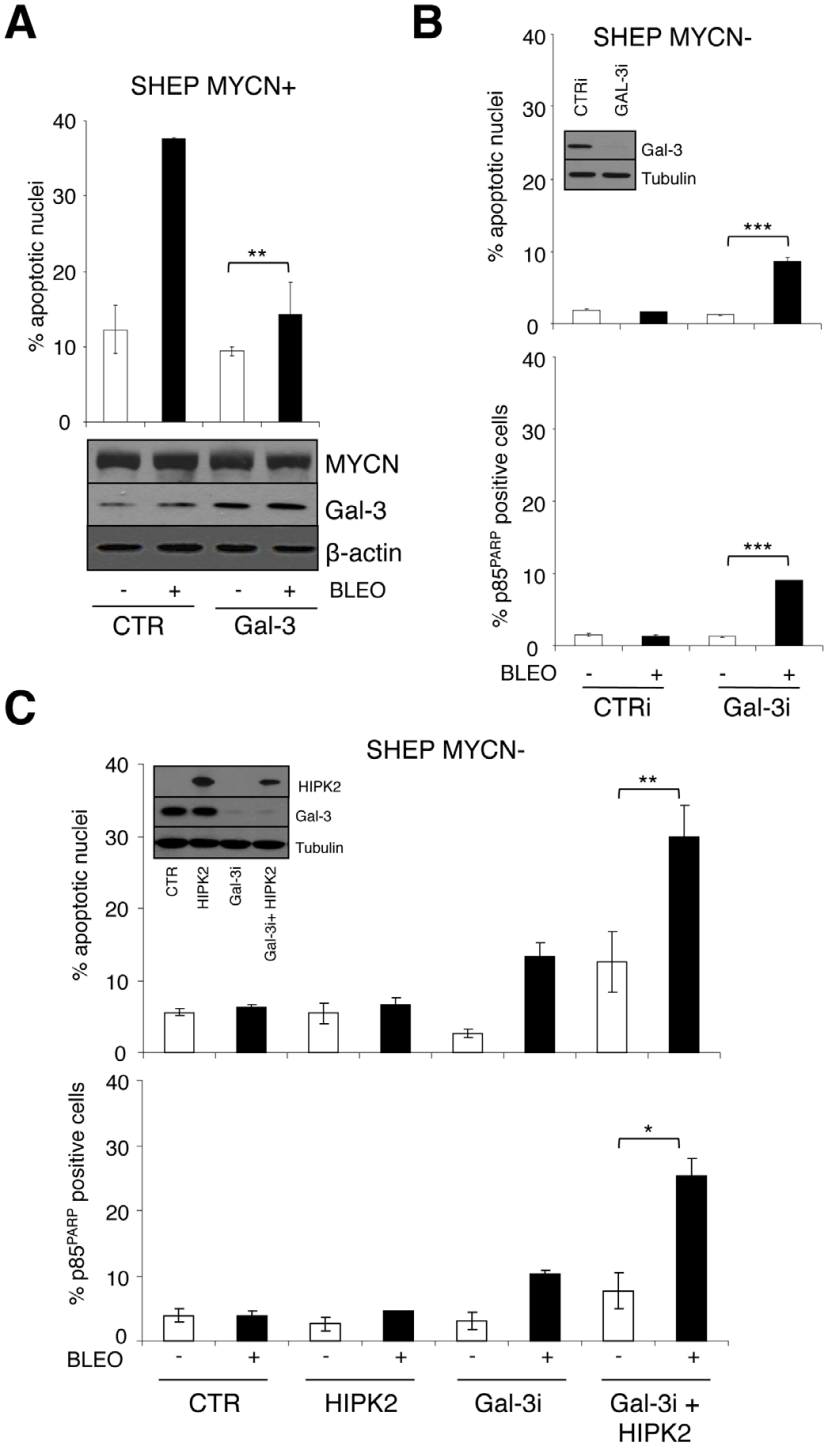
HIPK2 overexpression and Galectin-3 knock-down cooperate in sensitizing MNSC cells to apoptosis. A , Percentage of apoptotic nuclei in MYCN expressing SHEP cells following Gal-3 overexpression (immunoblot, lower panel) and bleomycin treatment. Significant differences in apoptosis fold induction were obtained between Gal-3 and pcDNA (CTR) transfected cells (**p<0.01). **B**, Gal-3 knock-down was achieved via transient transfection with RNAi duplexes in MNSC SHEP cells (inset). Analysis of bleomycin induced apoptosis is shown as percentage of apoptotic nuclei (upper panel) and p85^PARP^ positive cells (lower panel). Significant differences in apoptosis fold induction were obtained between Gal-3i and CTRi transfected cells (***p<0.0001). **C**, HIPK2 overexpression and Gal-3 knock-down were obtained in MNSC SHEP cells (inset) and the effect on apoptosis in basal condition and following bleomycin treatment was analyzed by counting the percentage of apoptotic nuclei (upper panel) and p85^PARP^ positive cells (lower panel). Significant differences in apoptosis fold induction were obtained in Gal-3i+HIPK2 transfected cells compared either to CTR or to Gal3i transfected cells (*p≤0.05; **p<0.01).

Primary Abs: anti-p85^PARP^ polyclonal Ab (Promega Corporation, Madison, WI), anti-Galectin-3 purified MoAb (Space Import & Export, Milan, Italy), MoAb anti-myc 9E10 (Santa Cruz Biotechnology, Santa Cruz, CA, USA).

Secondary Abs: Alexafluor 488 Goat anti-mouse igG (H+L), Alexafluor 594 Goat anti-rabbit IgG (H+L) (Invitrogen, Molecular Probes), Cy3 conjugated Affini Pure Donkey anti-mouse IgG (H+L) (Jackson ImmunoResearch Laboratories, West Grove, PA, USA).

### RNA Preparation and Quantitative Reverse Transcription-PCR

Total RNA extraction was carried out with TRIzol reagent (Invitrogen). For quantitative reverse transcription-PCR (Q-RT-PCR), total RNA (1 µg) was reverse transcribed using Gene Amp kit (Applied Biosystems, Warrington, UK) and subjected to PCR amplification using SYBR Green PCR Master Mix (Applied Biosystems) using an ABI Prism 7900 sequence detector (Applied Biosystems) as described [37]. Primer sequences were as follows:

hGAPDH forward, 5′-AGCAATGCCTCCTGCACCACCAAC-3′

hGAPDH reverse, 5′-CCGGAGGGGCCATCCACAGTCT-3′

hGalectin-3 forward, 5′-TCCACTTTAACCCACGCTTC-3′

hGalectin-3 reverse, 5′- TCTTCCCTTCCCCAGTTATT-3′

All amplification reactions were performed at least in duplicate and averages of threshold cycles were used to interpolate standard curves and calculate transcript amount using the SDS version 2.3 software (Applied Biosystems).

**Figure 4:**
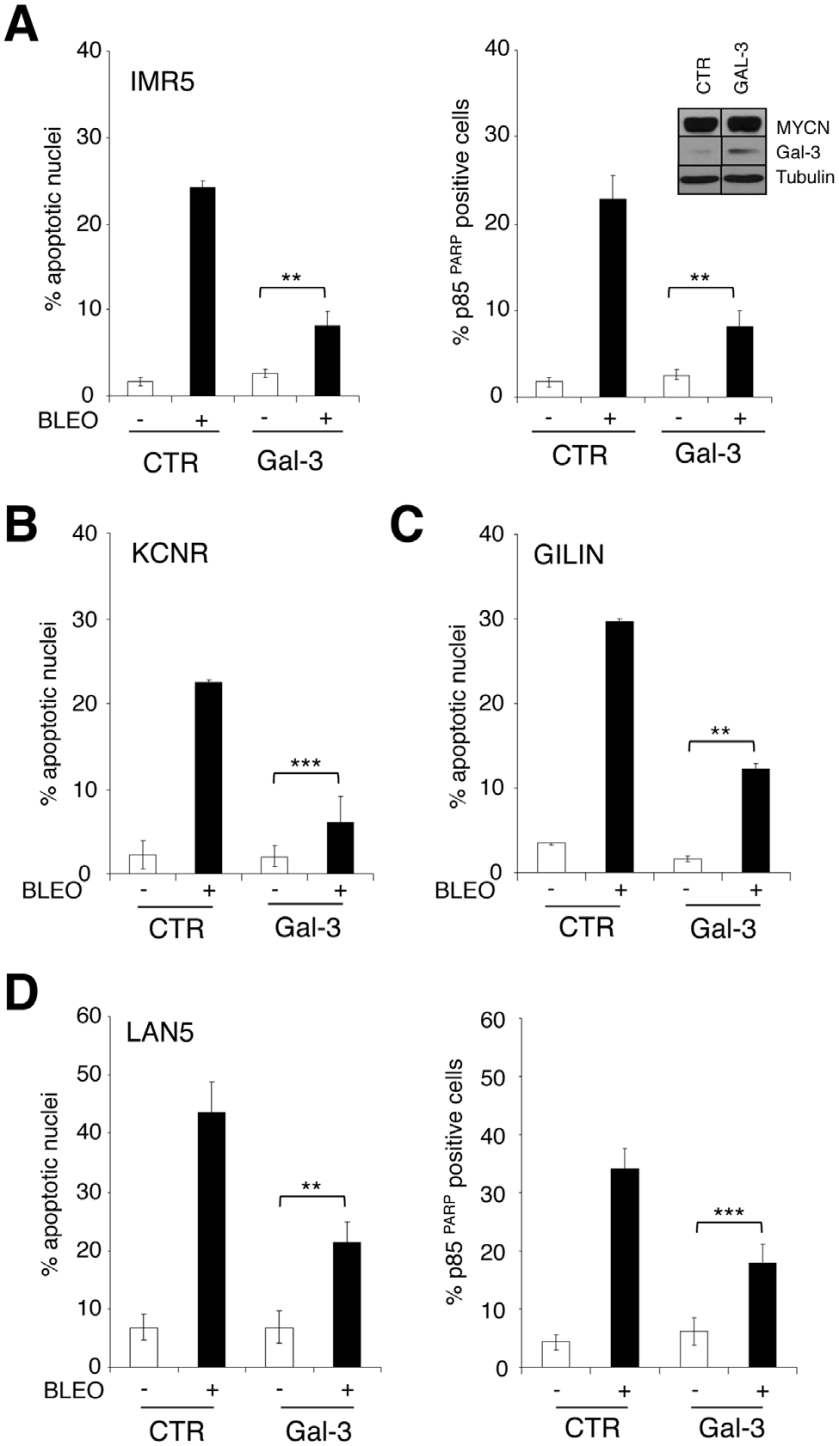
Galectin-3 overexpression protects MNA cells from apoptosis. Effect of transient Gal-3 overexpression (inset) on bleomycin-induced apoptosis in IMR-5 (**A**), KCNR (**B**) and GILIN (**C**) cells shown as percentage of apoptotic nuclei and/or p85^PARP^ positive cells. **D**, The same analysis was performed on three distinct LAN5 stable clones overexpressing Gal-3 compared to three control clones. Data are represented as averages (+/− standard deviations) of the raw data obtained (see Fig. S2). Significant differences in apoptosis fold induction were obtained between Gal-3 and CTR transfected cells (** p<0.01; *** p<0.0001).

### Protein Extraction, Subcellular Fractionation and Immunoblot

Total protein extracts were obtained in RIPA buffer (50 mM Tris pH 8, 150 mM NaCl, 0.5% Sodiodeoxycolate, 0.1% SDS, 1% NP40, 0.001 M EDTA and a mix of protease inhibitors). Fractionation of nuclei and cytoplasm from cultured cells was performed as described [27]. Fractionation of mitochondria and cytosol was performed as described [38] except that, following centrifugation at 10,000 *g* for 15 min at 4°C, the pellets were washed in 5 volumes of lysis buffer containing 250 mM sucrose and further centrifuged at 10,000 *g* for 15 min at 4°C to reduce cytosolic protein contaminations.

Total protein extracts (30 µg/sample) and subcellular fractions were separated by SDS-PAGE and blotted onto nitrocellulose membrane (PerkinElmer, Waltham, MA, USA). Membranes were blocked with 5% nonfat dry milk and incubated with primary Abs at the appropriate dilutions. Abs were as follows: polyclonal Ab anti-p85^PARP^ (Promega Corporation); mouse anti-p53 (DO-I), mouse anti-MYCN and mouse anti-β-tubulin MoAbs and goat anti-β-actin, and rabbit polyclonal anti-p38 (C20) Ab (Santa Cruz Biotechnology); mouse anti-c-Myc MoAb (Sigma Aldrich); rat anti-Galectin-3 purified monoclonal antibody (Space Import & Export); rabbit anti-HIPK2 polyclonal Ab (kindly provided by Prof. M.L. Schmitz), mouse monoclonal anti-cytochrome c Ab (Pharmingen). Immunoreactive bands were visualized by enhanced chemoluminescence (Perkin Elmer).

### Constructs, Transfections and RNA Interference

The sh-RNA interference pSUPER-HIPK2, the pEGFP-HIPK2 and the pcDNA3.1-galectin-3 vectors were previously described [39], [40], [41]. For Gal-3 stable transfection, PCR amplified human Gal-3 coding sequence was cloned in frame with the myc tag in the pcDNA3.1(−)/Myc-His A vector (Invitrogen). In transfection experiment the empty pcDNA3.1(−)/Myc-His A vector was used as mock control. IMR5, IMR32, U2OS and SHEP Tet21/N cells were transfected with Lipofectamine 2000 reagent (Invitrogen) according to the manufacturer’s instructions. KCNR, LAN5 and GILIN cells were transfected by electroporation with Nucleofector Solution V, in a Nuclefector II (Amaxa Byosistems, Gaithersburg, MD, USA). For stable integration, LAN5 cells were transfected with either the Gal-3 expression vector or the empty vector, selected in the presence of 400 µg/ml geneticin (G418, Sigma- Aldrich) and ten representative clones were isolated from each transfection. After Gal-3 expression analysis, three clones expressing different levels of Gal-3 were chosen for further analysis.

Gal-3 and MYCN knock down was obtained with siRNA interfering oligos (50 nM; Sigma-Aldrich) transfected by Dharmafect 2 Reagent (ThermoScientific, Dharmacon Rnai Technologies, UK) according to the manufacturer’s protocol. siRNA sequences were as follows:

Gal-3 sense, 5′-CAGAAUUGCUUUAGAUUUC-3′;

Gal-3 antisense 5′-GAAAUCUAAAGCAAUUCUG-3′;

Gal-3 no-target control sense, 5′-GCUUCAUUUAAGGUCAAUU -3′;

Gal-3 no-target control antisense, 5′-AAUUGACCUUAAAUGAAGC -3′.

MYCN sense, 5′-GUAUUAGACUGGAAGUUCA-3′;

MYCN antisense 5′-UGAACUUCCAGUCUAAUAC-3′);

Control no-target for MYCN was MISSION siRNA Universal Negative Control (SIC001, Sigma-Aldrich).

**Figure 5:**
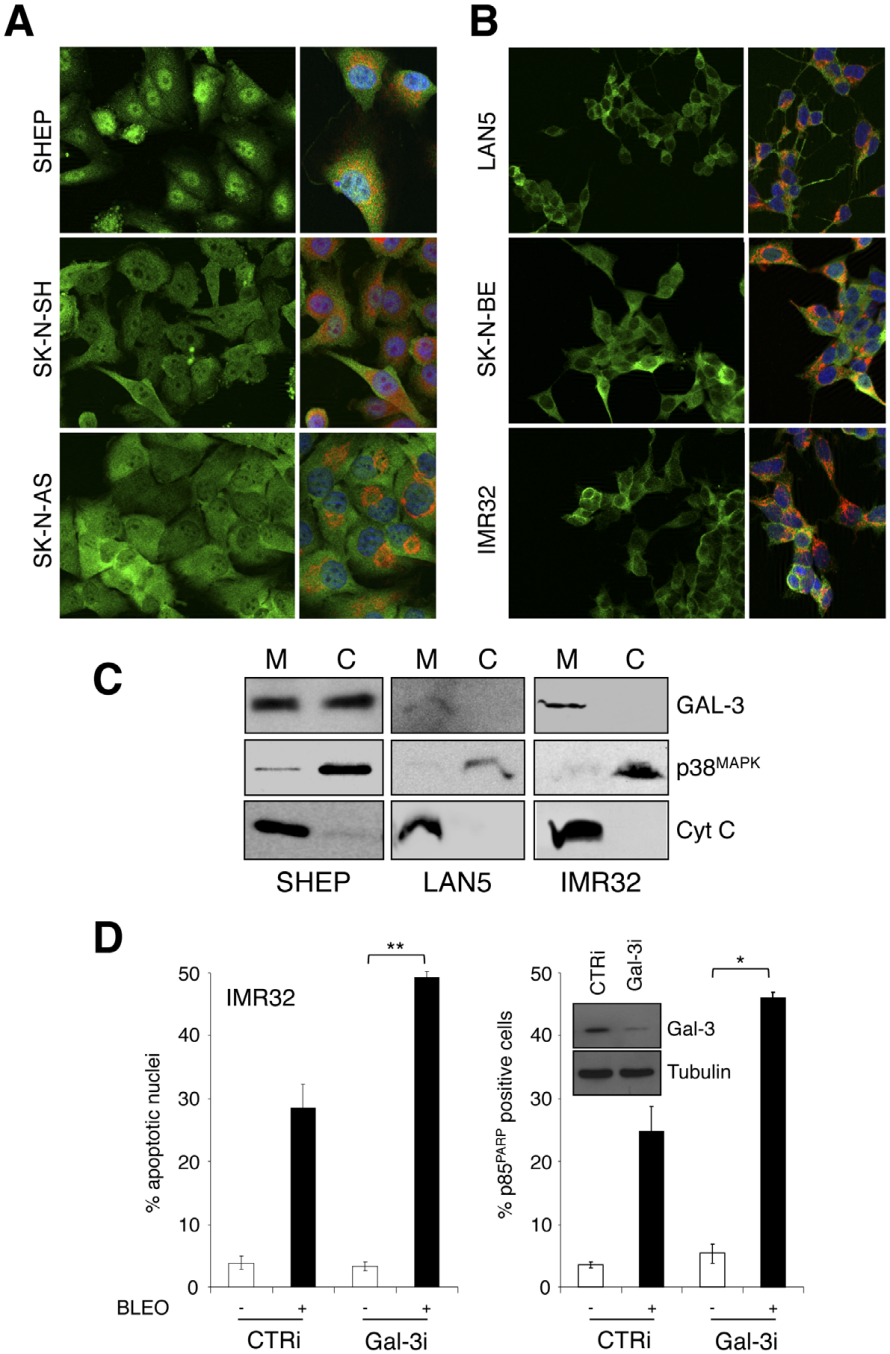
Galectin-3 intracellular localization in NB cells. To address Gal-3 localization, immunofluorescent analysis was performed on fixed MNSC (**A**) and MNA (**B**) NB cell lines; for each cell type Gal-3 immunostaining alone (left panels) and double staining with the MitoTracker (right panels) are shown. **C**, Immunoblot analysis of Gal-3 content in cell equivalent amounts of mitochondrial (M) and cytosolic (C) extracts from the indicated NB cell lines; p38^MAPK^ and Cyt-c were used as controls for cytosolic and mitochondrial enrichment, respectively. **D**, Effect of Gal-3 repression by RNAi (inset) on bleomycin-induced apoptosis in IMR32 NB cells shown as percentage of apoptotic nuclei and p85^PARP^ positive cells. Significant differences in apoptosis fold induction were obtained between Gal-3i and CTRi transfected cells (*p≤0.05; **p<0.01).

### NB Tumor Samples

Tumor samples from primary lesions were obtained from 25 children with previously untreated neuroblastoma admitted at the Department of Pediatrics, La Sapienza University. Institutional written informed consent was obtained from the patient’s parents or legal guardians and the study underwent review and approval by the Ethics Committee of “Policlinico Umberto I”. Each sample was characterized for MYCN amplification by Southern blot as previously described [42], [43].

## Results

### HIPK2 is Necessary but not Sufficient for the Induction of the Apoptosis-sensitive Phenotype by MYCN

MYCN sensitizes neuroblastoma cells to apoptosis [10], [44] by upregulating the HIPK2-p53 pathway [13], [20]. Consistently, p53 and HIPK2 depletion via RNA interference (RNAi) impairs apoptosis induced by DNA damaging drugs and p53 reactivating compounds in MNA and MYCN overexpressing cells [13], [19], [20] (see also Fig. 1A). Thus, in the presence of WT p53, HIPK2 upregulation by MYCN is required to induce the apoptosis-sensitive phenotype. However, HIPK2 expression did not induce apoptosis nor sensitized MNSC SHEP NB cells to bleomycin, while it was clearly sufficient to lead U2OS osteosarcoma cells to death (Fig. 1B upper panel). This suggests that MYCN should regulate additional mechanism/s collaborating with HIPK2 in order to sensitize NB cells to apoptosis. Among the potential candidates, we turned our attention on Galectin-3 (Gal-3), a multitalented protein involved in resistance to chemotherapeutic drugs [26] known to be functionally linked to the HIPK2-p53 circuitry [30], [31], [32]. Interestingly, Gal-3 was strongly expressed in the HIPK2 resistant SHEP cells as compared to the HIPK2 sensitive U2OS cells (Fig. 1B lower panel).

**Figure 6:**
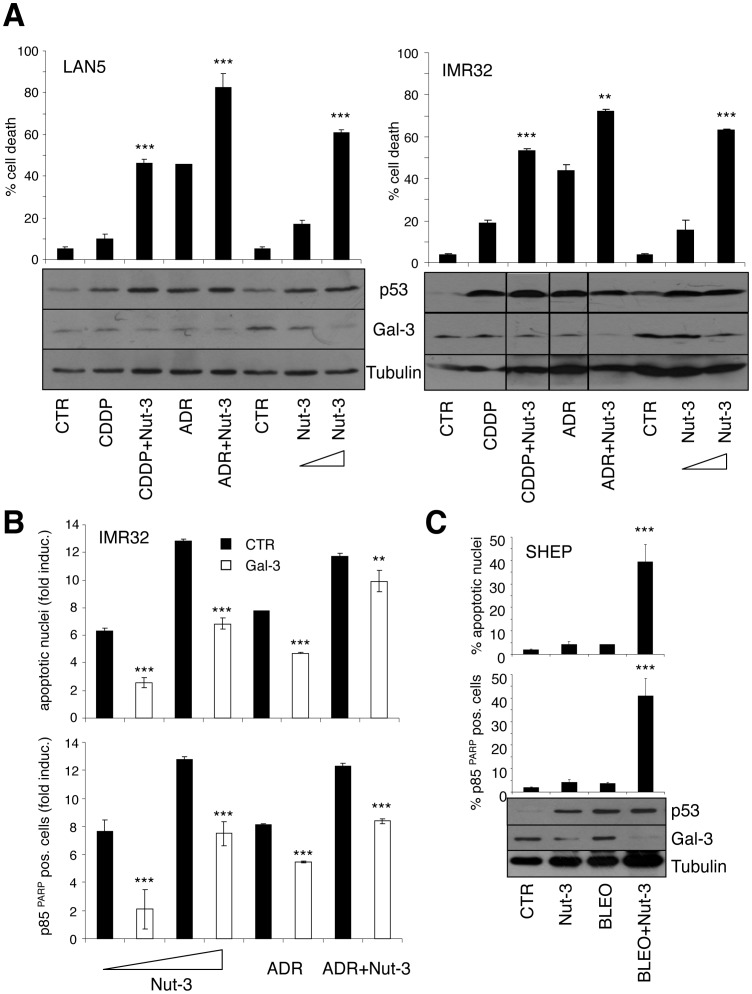
Nutlin-3 efficiently induces cell death and cooperates with clastogenic drugs by downregulating Gal-3 in MNA NB cells. **A**, Cell death induced by either cis-platin (CDDP, 1 µM), Adriamycin (ADR, 0.1 µM), Nut-3 (2 or 10 µM) or combination of Nut-3 (2 µM) with CDDP or ADR, was measured by Tripan blue-exclusion test (upper panel) in the indicated MNA NB cell lines and the expression of the indicated proteins was assessed by immunoblot (lower panel). Significant differences in cell death were obtained between Nut-3 treated samples versus the corresponding untreated controls (CTR) and/or single drug treated samples (**p<0.01; ***p<0.0001). B, Effect of Gal-3 overexpression on apoptosis induced by ADR and Nut-3 in IMR32 cells. Data are represented as averages (+/− standard deviations) of the apoptosis fold induction compared to untreated controls. Significant differences in apoptosis fold induction were obtained between Gal-3 and CTR transfected cells (**p<0.01; ***p<0.0001). C, Effects of Nut-3 (16 µM) on bleomycin induced apoptosis and Gal-3 expression in MNSC SHEP cells. Significant differences in apoptosis fold induction were obtained between bleomycin and Nut-3 treated samples versus bleomycin-only treated samples (***p<0.0001).

### MYCN Represses Gal-3 Expression

The analysis of a panel of NB cell lines and tumor samples revealed that Gal-3 mRNA is expressed at easily detectable levels in most samples. However its average expression was substantially lower in MNA compared to MNSC tumor samples and cell lines (Fig. 2A and Fig. S1). Consistently, Gal-3 protein is very little expressed in most MNA cell lines as compared with MNSC cells, with few exceptions (Fig. 2B). To evaluate whether MYCN might control Gal-3 expression we used two largely validated models of exogenous MYCN expression: the tetracycline inducible MYCN overexpressing SHEP Tet21/N cells [35], [45] and the MYCN stable transfectant SK-N-MYC [46]. Both cell systems showed that MYCN reduces Gal-3 at the mRNA and protein levels (Fig. 2C). Conversely, MYCN depletion by RNAi led to increased Gal-3 expression in the MNA and p53-null LAN1 cells (Fig. 2D). Overall these data indicate that MYCN impairs Gal-3 expression and raise the hypothesis that also Gal-3 repression (in addition to HIPK2 increase) might be required for the induction of the apoptosis-sensitive phenotype by MYCN.

### Coordinated Gal-3 Depletion and HIPK2 Overexpression Recapitulate the Apoptosis-sensitive Phenotype Induced by MYCN

To test whether Gal-3 repression by MYCN is involved in sensitization to apoptosis we overexpressed or depleted Gal-3 in MYCN expressing SHEP Tet21/N cells or in MYCN-repressed cells, respectively. Interestingly, increased Gal-3 expression protected MYCN expressing cells from bleomycin-induced apoptosis (Fig. 3A) and Gal-3 knock-down by RNAi modestly, but reproducibly, sensitized SHEP cells to bleomycin-induced apoptosis (Fig. 3B). Most importantly, bleomycin efficiently induced apoptosis in SHEP cells overexpressing HIPK2 and depleted of Gal-3 (Fig. 3C) to a level similar to that operated by MYCN overexpression (Fig. 3A). Therefore HIPK2 induction and Gal-3 repression are necessary and sufficient to sensitize NB cells to apoptosis and to fully recapitulate the apoptosis-sensitive phenotype induced by MYCN. To further support this concept, we analyzed the effect of transient Gal-3 transfection in multiple MNA cell lines (IMR5, KCNR, GILIN) displaying low levels of endogenous Gal-3. Indeed, Gal-3 overexpression impaired bleomycin induced apoptosis in all cell lines tested, as indicated by the reduction in apoptotic nuclei and/or in the rate of cells staining for the cleaved fragment of the PARP protein (Fig. 4A, B, C). Similar results were obtained in three single clones of LAN5 cells stably overexpressing variable levels of Gal-3 (Fig. 4D and Fig. S2).

### Gal-3 Localization in MNSC and MNA NB Cell Lines

Gal-3 is a multitalented protein whose activity is strictly linked to its subcellular localization (reviewed in [23], [25]). Its prevalent cytoplasmic versus nuclear localization confers a transformed phenotype to breast and thyroid cells, and is associated with a more advanced/metastatic tumor state and aggressive behavior [24], [41], [47]. Gal-3 nuclear export to the cytoplasm also appears to be functionally involved in protecting cells from drug-induced apoptosis and is regulated by the casein kinase 1 (CK1) -dependent phosphorylation of Ser^6^ [27]. Furthermore, Gal-3 can extensively localize in the mitochondria and counteract apoptosis by stabilizing mitochondrial membranes and inhibiting cytochrome c release [28], [29], [48]. Therefore we sought to investigate on Gal-3 subcellular localization in MNSC and MNA neuroblastoma cell lines. Interestingly, MNSC cells (SHEP, SK-N-SH, SK-N-AS and GICAN) clearly showed both nuclear and cytoplasmic Gal-3 localization (Fig. 5A and Fig. S3A). Consistent with published data, inhibition of CK1 impaired Gal-3 nuclear export and sensitized SHEP cells to apoptosis (Fig. S3B), confirming that Gal-3 subcellular localization impacts on its antiapoptotic activity also in NB cells. Furthermore, double-staining experiments with the mitochondrial selective dye MitoTracker and the analysis of protein extracts enriched for cytosolic or mitochondrial proteins indicated a large localization of Gal-3 in the mitochondria of MNSC cells (Fig. 5A and 5C). In contrast, the MNA cells (LAN5, SK-N-BE, IMR32, KCNR, LAN1 and GILIN) showed an almost exclusive Gal-3 localization in the cytoplasm with very little or no staining in the nucleus (Fig. 5B and Fig. S3A). Interestingly, cytoplasmic Gal-3 staining largely overlapped the MitoTracker suggesting that even the low levels of Gal-3 expressed in the MNA cells might at least partially localize in the mitochondria (Fig. 5B). This conclusion was further supported by the analysis of protein extracts enriched for cytosolic or mitochondrial proteins (Fig. 5C). The different Gal-3 localization pattern between MNSC and MNA cells is not due to its different levels of expression but appears to be actively regulated, since overexpressed Gal-3 localization closely resembles that of the endogenous protein both in MNSC and MNA cell lines (not shown and Fig. S3C). Therefore, Gal-3 localization in the cytoplasm and mitochondria is consistent with its antiapoptotic role in NB cells and the reduced levels of Gal-3 expression in MNA versus MNSC cells are compatible with their increased sensitivity to apoptosis.

**Figure 7:**
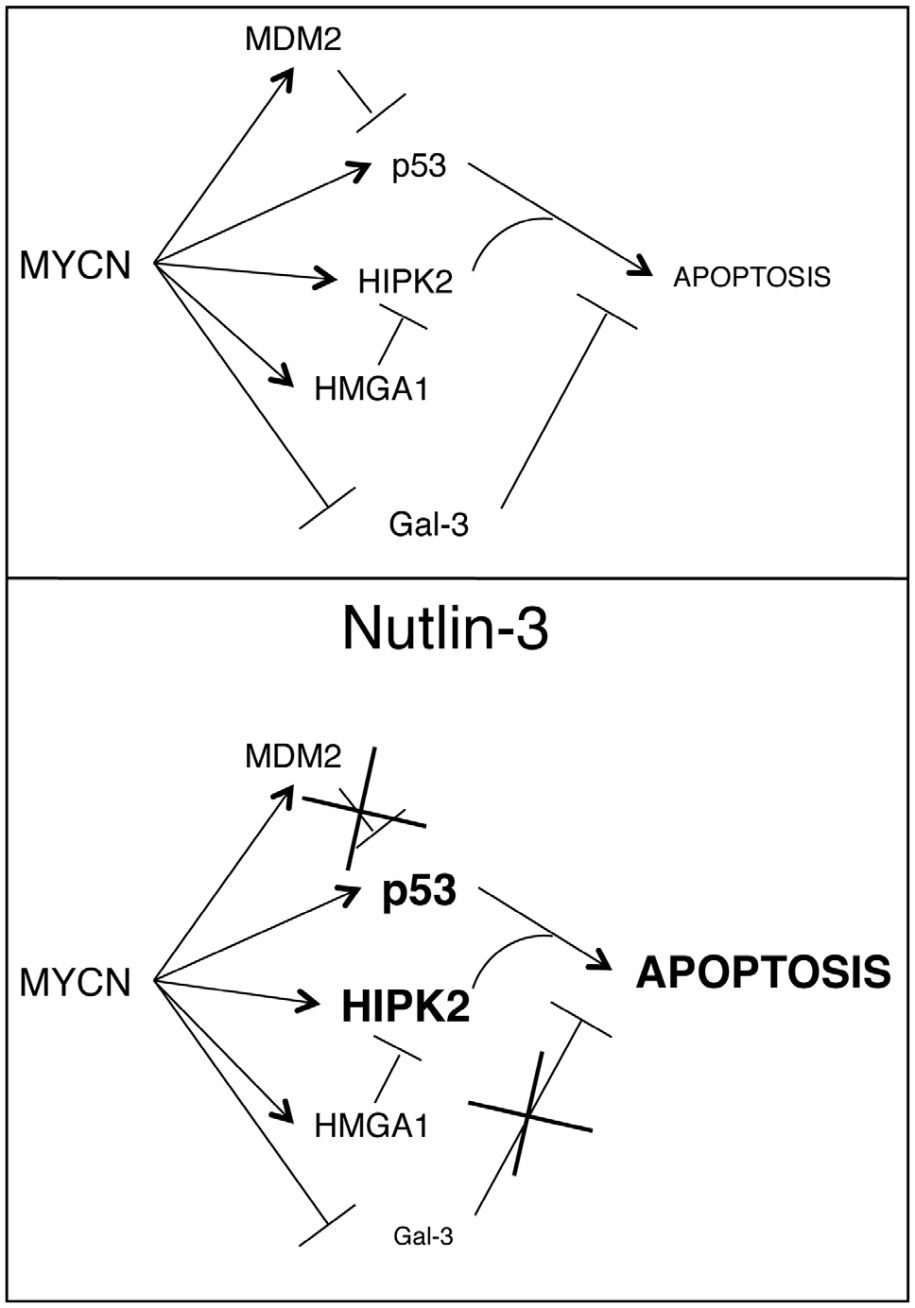
Regulation of the p53 proapoptotic pathway by MYCN and effects of Nut-3. MNA amplified and MYCN overexpressing cells are characterized by a delicate equilibrium between pro- and anti-apoptotic factors of the p53 pathway that are directly or indirectly regulated by MYCN. Unbalancing these factors may easily trigger apoptosis in this context. In particular Nut-3 is highly effective because it increases p53 expression and its S^46^ phosphorylation via HIPK2 induction and at the same time it represses Gal-3, in MNA NB cells.

### Gal-3 Protects MNA Neuroblastoma Cells from Apoptosis

Although the reduced level of Gal-3 expression generally observed in MNA cells is compatible with their increased sensitivity to apoptosis, Gal-3 is expressed in the cytoplasm and at least partially localized in the mitochondria also in these cells. To directly address whether Gal-3 might exert an antiapoptotic effect also in this context we knocked it down in the IMR32 MNA cells (that possess higher endogenous levels of Gal-3 compared to most other MNA cells, see Fig. 2). Gal-3 knock-down further sensitized IMR32 cells to apoptosis induced by bleomycin (Fig. 5D). These data indicate that, due to its peculiar subcellular localization pattern, the low amount of Gal-3 expressed in MNA NBs is still likely to contribute to the aggressive phenotype of this tumor subset and its repression might be required to enforce the pharmacological induction of apoptosis.

### Nutlin-3 Represses Gal-3 to Efficiently Induce Apoptosis in MNA Neuroblastoma Cells

Upon DNA damage, HIPK2 activated p53 can repress Gal-3 and its antiapoptotic activity [30]. However this does not seem to be the case in NB. Indeed, neither bleomycin, nor cis-platin (CDDP), nor adriamycin reduced Gal-3 expression in NB cells (Fig. 1, 3, 6). MDM2-p53 antagonists trigger cell death more efficiently than DNA damaging drugs and cooperatively with them in MNA NB cells, since they very efficiently engage the proapoptotic power of the HIPK2/p53 pathway by inducing HIPK2, in this cell type [13]. Thus, we questioned whether and how MDM2-p53 antagonists, such as Nut-3, would affect Gal-3 expression in MNA cells. As previously shown [13], at 10 µM concentration, Nut-3 induced cell death more efficiently than CDDP and adriamycin used at 1 µM and 0.1 µM concentration, respectively (Fig. 6A). Furthermore, suboptimal amounts (2 µM) of Nut-3 synergized with both drugs to induce cell death. Interestingly, Nut-3 repressed Gal-3 expression in both cell lines in a dose dependent manner (Fig. 6A). Together with the increased apoptotic effect, Nut-3 also decreased Gal-3 in co-treatment with CDDP and adryamicin. Furthermore, Gal-3 overexpression impaired apoptosis induced by Nut-3 alone or in combination with adriamycin in IMR32 and LAN5 MNA cells (Fig. 6B and Fig. S4), indicating that Gal-3 repression by Nut-3 is linked to its potent apoptotic activity on MNA NB cells. Consistent with these results, Gal-3 repression by Nut-3 also sensitized SHEP cells to bleomycin induced apoptosis (Fig. 6C).

## Discussion

The p53 oncosuppressive pathway is almost ubiquitously impaired in cancer, either due to mutations of p53 gene itself or to alterations of the numerous modulators of p53 activity [16]. Therefore, restoring p53 functions might represent an effective approach in the treatment of a broad range of human cancers and is capturing great interest and energies. In animal models, p53 restoration already proved to be extremely effective in promoting tumor regression [49], [50], [51] and several strategies aimed at restoring p53 function (i.e., p53 gene therapy, wild type p53 restoration via small molecole/MDM2 antagonists and mutant-p53 folding restoration) are being currently tested in clinical trials [52]. Since newly diagnosed human NBs are almost invariably p53 wild type and preclinical data indicate that NB cells are strikingly sensitive to Nut-3 in *in vitro* and in xenograft models [13], [15], translation of the p53 restoration therapy in the treatment of high-risk NB patients urges. Thus, a better understanding of the biological processes governing the sensitivity of NB cells to MDM2-p53 antagonists and the potential identification of biomarkers of the p53 pathway are pivotal in tailoring this therapeutic approach to NB patients.

Studying the apparently paradoxical ability of the MYCN protoncogene to induce apoptosis or the apoptosis-sensitive phenotype in neuroblastoma cells provided important clues on the activity of MDM2/p53 antagonists and on their potential pharmacological use in NB therapy. Indeed, a particular regulation of the p53 pathway seems to occur in MNA NBs, in which both pro- and anti-apoptotic factors appear to be directly or indirectly controlled by MYCN [13], [20], [53], [54], [55], [56], [57], as shown in Fig. 7. In particular, MYCN induces p53 expression and accumulation of its proapoptotic activator kinase HIPK2, which is essential for the well-known sensitization to apoptosis induced by MYCN [13]. However, HIPK2 overexpression alone fails to sensitize MNSC NB cells to apoptosis, this suggesting that additional events must be required to recapitulate MYCN sensitizing effect. Here we showed that Gal-3, a potent inhibitor of apoptosis [26], modulates the HIPK2/p53 dependent cell death. Gal-3 is a ß-galactoside-binding lectin involved in a variety of biological processes including pre-mRNA processing, cell cycle progression, cell adhesion, angiogenesis and apoptosis [23]. The prevailing Gal-3 activity largely depends on the cell type and subcellular localization. Therefore, it is not surprising that either increased or even decreased levels of Gal-3 have been associated with more advanced tumor stages and/or reduced survival in different human cancers [58], [59], [60], [61], [62], [63]. Importantly, analysis of Gal-3 expression has already acquired diagnostic relevance in a few specific clinical settings, such as the preoperative characterization of thyroid nodules where it is widely used as a marker of malignancy [64], [65], [66].

Gal-3 role had never been investigated in NB cells. Here we have shown that Gal-3 is broadly expressed in human NB cell lines and tumors and is downregulated by MYCN at the mRNA and protein level. We recently reported that MYCN induces the expression of both p53 and HIPK2 [13] and HIPK2 activated p53 can repress Gal-3 transcription [30]. Thus MYCN might lead to Gal-3 repression via p53. However, we found relatively low levels of Gal-3 also in MNA and p53 mutant NB cell lines and reported Gal-3 increase upon MYCN knock-down in p53 deficient NB cells. Therefore, although preliminary evidence suggests that MYCN might regulate Gal-3 at least in part at the transcriptional level, this does not require p53. Additional work will shed light on the molecular mechanisms responsible for Gal-3 repression by MYCN. Most important, however, we found that in cooperation with HIPK2 overexpression, Gal-3 repression fully mimics MYCN ability to sensitize NB cells to apoptosis, switching on a spot-light on the complex regulation of Gal-3 as an important modulator of this pathway. In fact, besides being quantitatively regulated, Gal-3 protein is subjected to intense cellular trafficking. Depending on the cell context and its phosphorylation status, it might display prevalent or exclusive localization in the nucleus, the cytoplasm, the mitochondria, or it might be secreted into the extracellular matrix. Of interest, its subcellular localization appears strictly connected to its biological functions. For instance, its nuclear overexpression in prostate cancer cells leads to apoptosis and inhibition of cell cycle progression, while forcing its localization in the cytoplasm mostly protects cells from apoptosis [24]. Furthermore, predominant cytoplasmic localization has been detected in more advanced stages in several types of epithelial cancer ([24] and references therein) leading to the hypothesis that Gal-3 might exert an oncosuppressive role when it is localized in the nucleus versus a more oncogenic role when its cytoplasmic localization prevails. The pattern of localization we detected in human NB cell lines appears consistent with this model. Indeed, Gal-3 is diffusely distributed in the nucleus, cytosol and mitochondria in MNSC cells, which are modestly tumorigenic in nude mice [67], despite their apoptosis-resistant phenotype. In contrast, Gal-3 is substantially absent from the nucleus in MNA cell lines, consistent with them being very tumorigenic in nude mice and with the known aggressiveness of MNA NBs in humans. Interestingly, the low amount of Gal-3 expressed in MNA cells is largely concentrated in the mitochondria. This pattern does not depend on the reduced levels of Gal-3 expression and might be directly controlled by yet unknown functions of MYCN, since both endogenous and overexpressed Gal-3 show a similar distribution in MNA cell lines.

Although the general decrease of Gal-3 we detected in MNA appears to be connected to a generally higher sensitivity to apoptosis compared to MNSC cells, its prevalent localization in the mitochondria makes it still functionally relevant in the control of apoptosis. Indeed, Gal-3 repression by RNAi further sensitized MNA cells to cell death suggesting that pharmacological treatments aimed at repressing Gal-3 might enforce the induction of apoptosis in this context. Upon DNA damage, HIPK2 activated p53 was shown to repress Gal-3 [30]. However, bleomycin, adriamycin and CDDP failed to do so in NB cells. In contrast, we found that Nut-3 represses Gal-3 in order to most effectively kill MNA cells, either alone or in combination with chemotoxic drugs. Consistently, Gal-3 overexpression impairs apoptosis induced by Nut-3 alone, or in combination with adriamycin.

We previously reported that MDM2-p53 antagonists like Nut-3 effectively induce cell death and synergize with cytotoxic drugs in MNA NB cells by inducing both p53 and its proapoptotic kinase HIPK2, thus enhancing p53 phosphorylation at serine 46 and its commitment towards an apoptotic pathway [13]. Now we have added another piece to the puzzle and additional mechanistic explanation to the potent effects of MDM2-p53 antagonists in MNA NBs by showing that Gal-3 is an important source of resistance to p53-dependent apoptosis that can be relieved by Nut-3 (Fig. 7).

In conclusion, our data increase our understanding of MYCN-dependent apoptosis, provide additional molecular background to the activity of MDM2-p53 antagonists and further strengthen the need to test them in the treatment of MNA NB. Finally, the newly identified role of Gal-3 in NB cell apoptosis identifies a new potential biomarker for the tailored p53 reactivation therapy in patients with high-risk NB.

## Supporting Information

Figure S1 **Gal-3 is differentially expressed in MNSC vs MNA NB tumor samples and cell lines.** Analysis of Gal-3 mRNA expression in NB cell lines (A) and primary human tumors (B) by Q-RT-PCR. Asterisks indicate MNA tumor samples.
(PDF)

Figure S2 **Gal-3 overexpression in LAN5 MNA cells.** Three distinct LAN5 cell clones stably expressing different levels of Gal-3 and three control clones (immunoblot in the upper panel) were chosen to illustrate the effects of Gal-3 overexpression on bleomycin-induced apoptosis shown as percentage of apoptotic nuclei and/or p85^PARP^ positive cells (raw data of experiments shown in Fig. 4D).
(PDF)

Figure S3 **Gal-3 intracellular localization in NB cells.** Immunofluorescent analysis showing Gal-3 localization in MNSC (GICAN) and MNA (LAN1, GILIN, KCNR) NB cell lines (A) and in LAN5 stable clone number 6 (C). B, Impairment of Gal-3 nuclear export by inhibition of CK1 with the CK1-inhibitor D4476 (CK1i, immunoblot in the inset) sensitizes SHEP cells to bleomycin induced cell death as measured by Tripan blue-exclusion test. Significant differences in cell death fold induction were obtained between bleomycin+CK1i treated samples versus bleomycin-only treated samples (***p<0.0001).
(PDF)

Figure S4 **Gal-3 overexpression protects MNA cells from apoptosis.** A, The effect of Gal-3 overexpression on apoptosis induced by ADR (0.1 µM), Nut-3 (2 or 10 µM) or combination of Nut-3 (2 µM) with ADR in IMR32 cells is shown as percentage of apoptotic nuclei and p85^PARP^ positive cells. B, Effect of Gal-3 overexpression on apoptosis induced by ADR and Nut-3 in LAN5 stable clones shown as percentage of apoptotic nuclei and p85^PARP^ positive cells.
(PDF)
