# Supplementary figures and images for: Galectin-3 Impairment of MYCN-Dependent Apoptosis-Sensitive Phenotype Is Antagonized by Nutlin-3 in Neuroblastoma Cells
Source: PLoS One. 2012 Nov 9;7(11):e49139. doi: 10.1371/journal.pone.0049139 (PMC3494673; doi:10.1371/journal.pone.0049139)

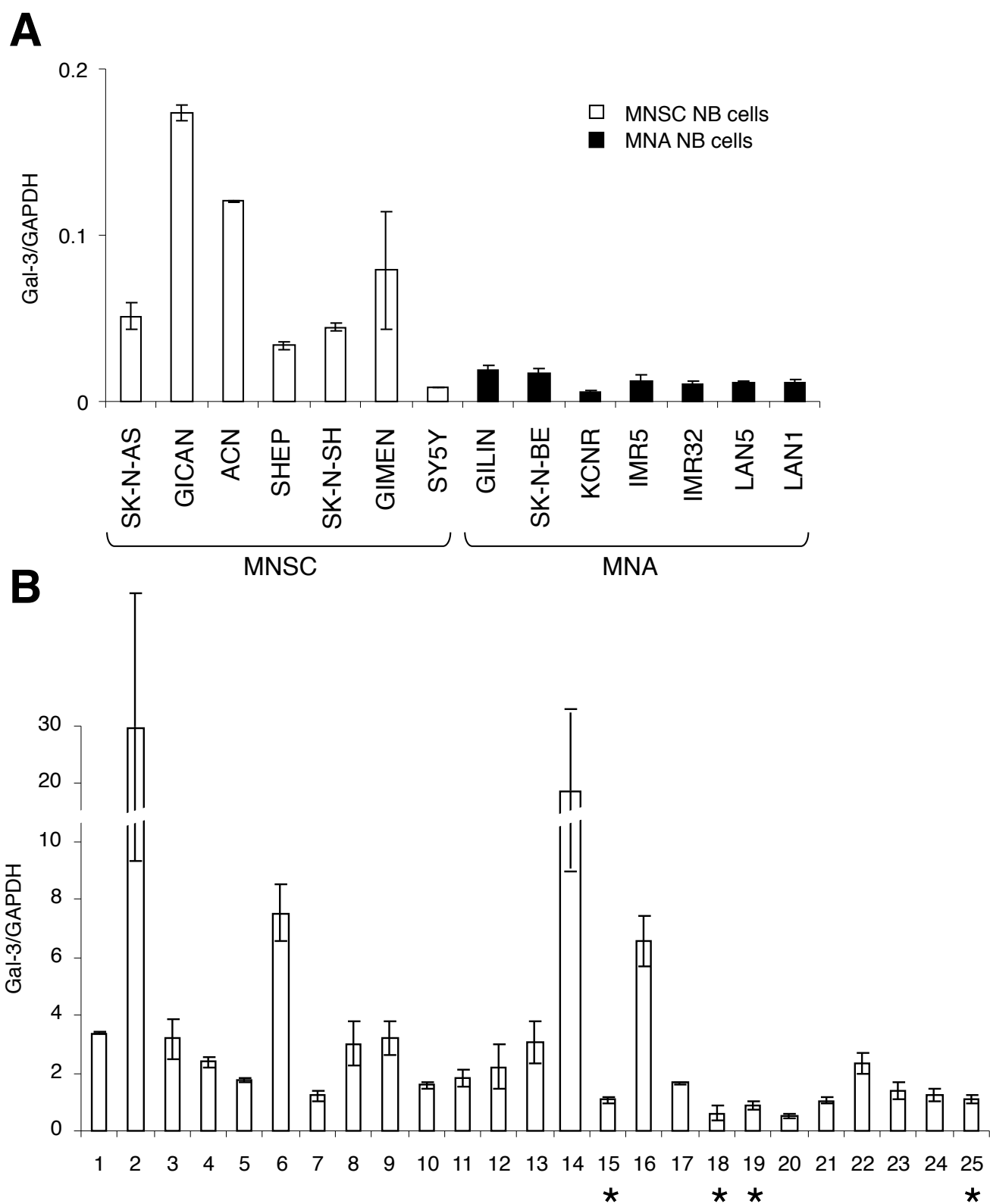

**Fig. S1**

Supplement: Figure S1 — Gal-3 is differentially expressed in MNSC vs MNA NB tumor samples and cell lines. Analysis of Gal-3 mRNA expression in NB cell lines (A) and primary human tumors (B) by Q-RT-PCR. Asterisks indicate MNA tumor samples. (PDF) [file pone.0049139.s001.pdf]

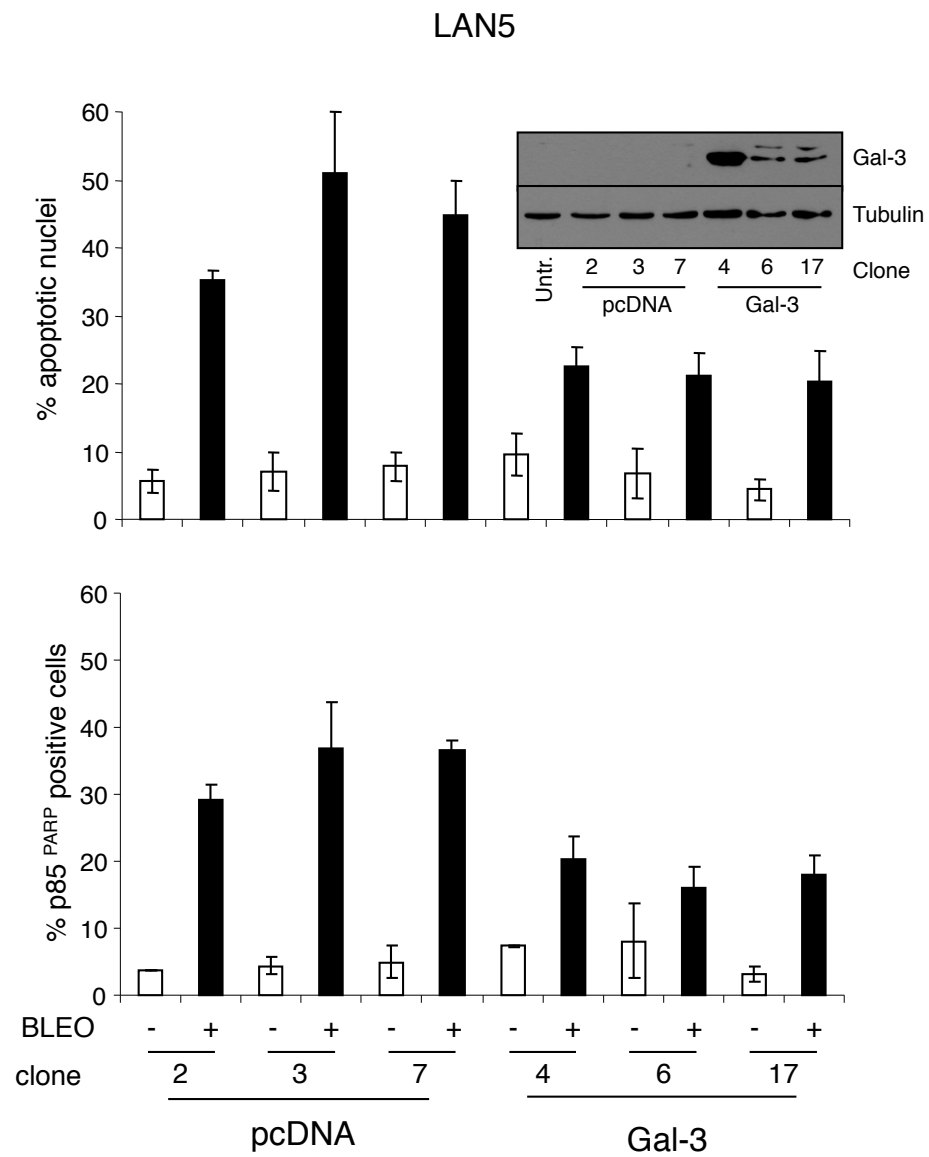

**Fig. S2**

Supplement: Figure S2 — Gal-3 overexpression in LAN5 MNA cells. Three distinct LAN5 cell clones stably expressing different levels of Gal-3 and three control clones (immunoblot in the upper panel) were chosen to illustrate the effects of Gal-3 overexpression on bleomycin-induced apoptosis shown as percentage of apoptotic nuclei and/or p85PARP positive cells (raw data of experiments shown in Fig. 4D). (PDF) [file pone.0049139.s002.pdf]

**A**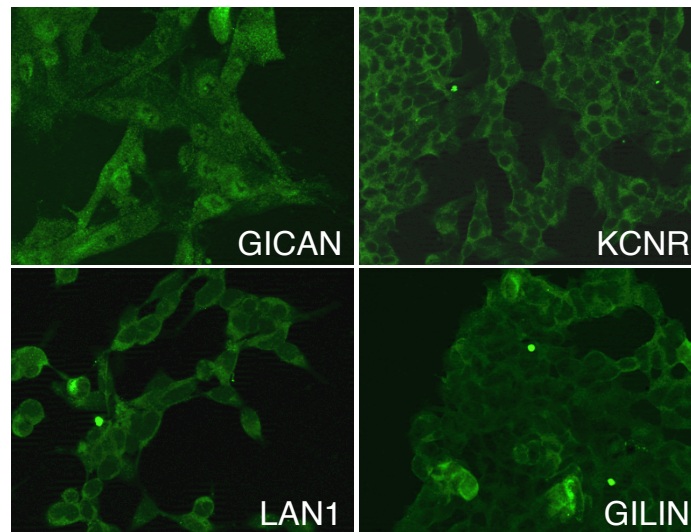**B**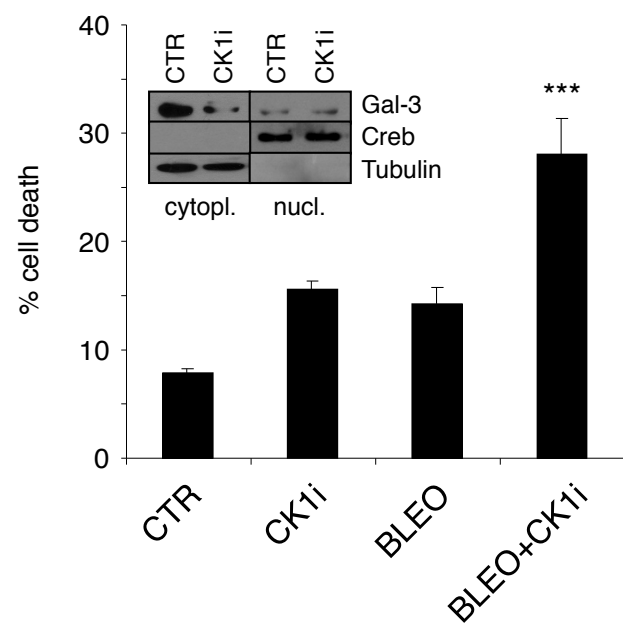**C**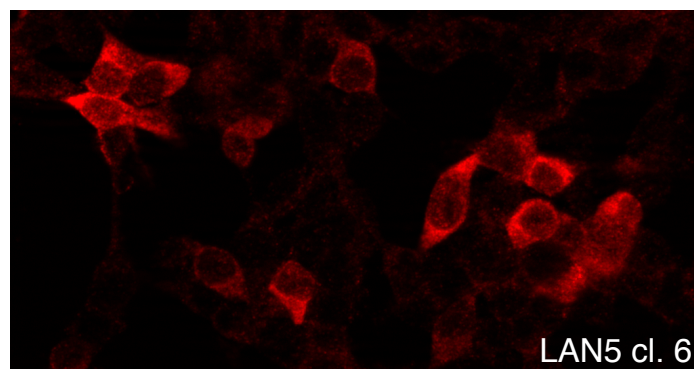

**Fig. S3**

Supplement: Figure S3 — Gal-3 intracellular localization in NB cells. Immunofluorescent analysis showing Gal-3 localization in MNSC (GICAN) and MNA (LAN1, GILIN, KCNR) NB cell lines (A) and in LAN5 stable clone number 6 (C). B, Impairment of Gal-3 nuclear export by inhibition of CK1 with the CK1-inhibitor D4476 (CK1i, immunoblot in the inset) sensitizes SHEP cells to bleomycin induced cell death as measured by Tripan blue-exclusion test. Significant differences in cell death fold induction were obtained between bleomycin+CK1i treated samples versus bleomycin-only treated samples (***p<0.0001). (PDF) [file pone.0049139.s003.pdf]

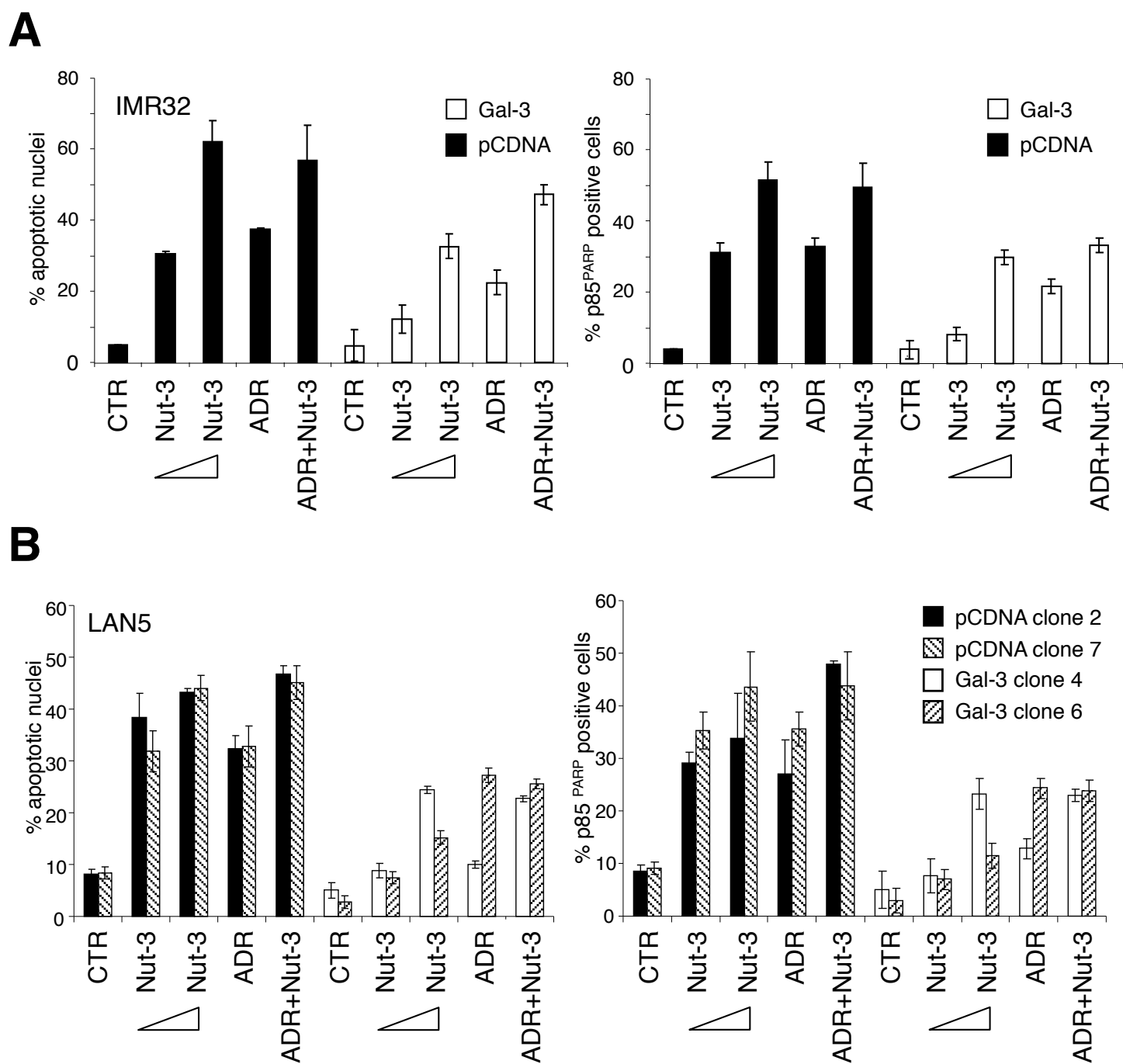

Fig. S4

Supplement: Figure S4 — Gal-3 overexpression protects MNA cells from apoptosis. A, The effect of Gal-3 overexpression on apoptosis induced by ADR (0.1 µM), Nut-3 (2 or 10 µM) or combination of Nut-3 (2 µM) with ADR in IMR32 cells is shown as percentage of apoptotic nuclei and p85PARP positive cells. B, Effect of Gal-3 overexpression on apoptosis induced by ADR and Nut-3 in LAN5 stable clones shown as percentage of apoptotic nuclei and p85PARP positive cells. (PDF) [file pone.0049139.s004.pdf]
